# Could texture features from preoperative CT image be used for predicting occult peritoneal carcinomatosis in patients with advanced gastric cancer?

**DOI:** 10.1371/journal.pone.0194755

**Published:** 2018-03-29

**Authors:** Hae Young Kim, Young Hoon Kim, Gabin Yun, Won Chang, Yoon Jin Lee, Bohyoung Kim

**Affiliations:** 1 Department of Radiology, Seoul National University Bundang Hospital, Seoul National University College of Medicine, Institute of Radiation Medicine, Seoul National University Medical Research Center, Seoul City, South Korea; 2 Division of Biomedical Engineering, Hankuk University of Foreign Studies, Seoul City, South Korea; Memorial Sloan Kettering Cancer Center, UNITED STATES

## Abstract

**Purpose:**

To retrospectively investigate whether texture features obtained from preoperative CT images of advanced gastric cancer (AGC) patients could be used for the prediction of occult peritoneal carcinomatosis (PC) detected during operation.

**Materials and methods:**

51 AGC patients with occult PC detected during operation from January 2009 to December 2012 were included as occult PC group. For the control group, other 51 AGC patients without evidence of distant metastasis including PC, and whose clinical T and N stage could be matched to those of the patients of the occult PC group, were selected from the period of January 2011 to July 2012. Each group was divided into test (n = 41) and validation cohort (n = 10). Demographic and clinical data of these patients were acquired from the hospital database. Texture features including average, standard deviation, kurtosis, skewness, entropy, correlation, and contrast were obtained from manually drawn region of interest (ROI) over the omentum on the axial CT image showing the omentum at its largest cross sectional area. After using Fisher's exact and Wilcoxon signed-rank test for comparison of the clinical and texture features between the two groups of the test cohort, conditional logistic regression analysis was performed to determine significant independent predictor for occult PC. Using the optimal cut-off value from receiver operating characteristic (ROC) analysis for the significant variables, diagnostic sensitivity and specificity were determined in the test cohort. The cut-off value of the significant variables obtained from the test cohort was then applied to the validation cohort. Bonferroni correction was used to adjust *P* value for multiple comparisons.

**Results:**

Between the two groups, there was no significant difference in the clinical features. Regarding the texture features, the occult PC group showed significantly higher average, entropy, standard deviation, and significantly lower correlation (*P* value < 0.004 for all). Conditional logistic regression analysis demonstrated that entropy was significant independent predictor for occult PC. When the cut-off value of entropy (> 7.141) was applied to the validation cohort, sensitivity and specificity for the prediction of occult PC were 80% and 90%, respectively.

**Conclusion:**

For AGC patients whose PC cannot be detected with routine imaging such as CT, texture analysis may be a useful adjunct for the prediction of occult PC.

## Introduction

Peritoneal carcinomatosis (PC) in patients with advanced gastric cancer (AGC) is generally regarded as an incurable systemic disease with poor prognosis, for which surgery is reserved only for palliative or cytoreductive purposes, and for which multimodal approaches including systemic chemotherapy may be attempted [1–3]. However, recent advances in chemotherapy regimen, targeted therapy, and use of intraperitoneal chemotherapy combined with cytoreductive surgery have shown promise for improvement of the survival rate [4–6]. Thus, knowledge of the presence of PC prior to surgery would aid in selection of patients for optimal therapy: patients without PC would receive surgery while patients with PC would receive intraperitoneal chemotherapy combined with cytoreductive surgery.

Computed tomography (CT) is generally used for preoperative staging work up of gastric cancer, and it shows promising diagnostic accuracy for detection of hepatic and distant nodal metastasis [7,8]. On the other hand, presence of PC is often missed with CT, which shows decreased sensitivity for tumor implants with a size of 1 cm or smaller [9]. In fact, a previous research using multidetector CT (MDCT) showed limited sensitivity of about 28% for the detection of PC in patients with AGC [10].

Intratumoral heterogeneity, which reflects variation in the tumor cell differentiation, cellularity, angiogenesis, and extracellular matrix, is generally accepted as a typical finding of malignancy [11]. Texture analysis, which can be applied to diverse imaging modalities including radiography, ultrasound, CT, magnetic resonance imaging, and positron emission tomography, is a non-invasive technique that quantifies tumoral heterogeneity by evaluating spatial variation in gray-level intensities in images [11]. This technique has received attention especially in the field of oncologic imaging for various malignant tumors, where measurement of intratumoral heterogeneity could be helpful in the prediction of tumor grade, treatment response, and prognosis [12–16]. However, to the best of our knowledge, there has been no previous report on the role of texture analysis in the detection of PC. Therefore, the purpose of this study was to retrospectively investigate whether texture features obtained from preoperative CT images of AGC patients could be used for the prediction of occult PC, defined as PC that could not be detected preoperatively with conventional staging work-up including CT until its confirmation during the operation.

## Materials and methods

This study was approved by the Institutional Review Board of Seoul National University Bundang Hospital (B-1705-396-108) and requirement of informed consent was waived.

### Patient population

**Fig 1:**
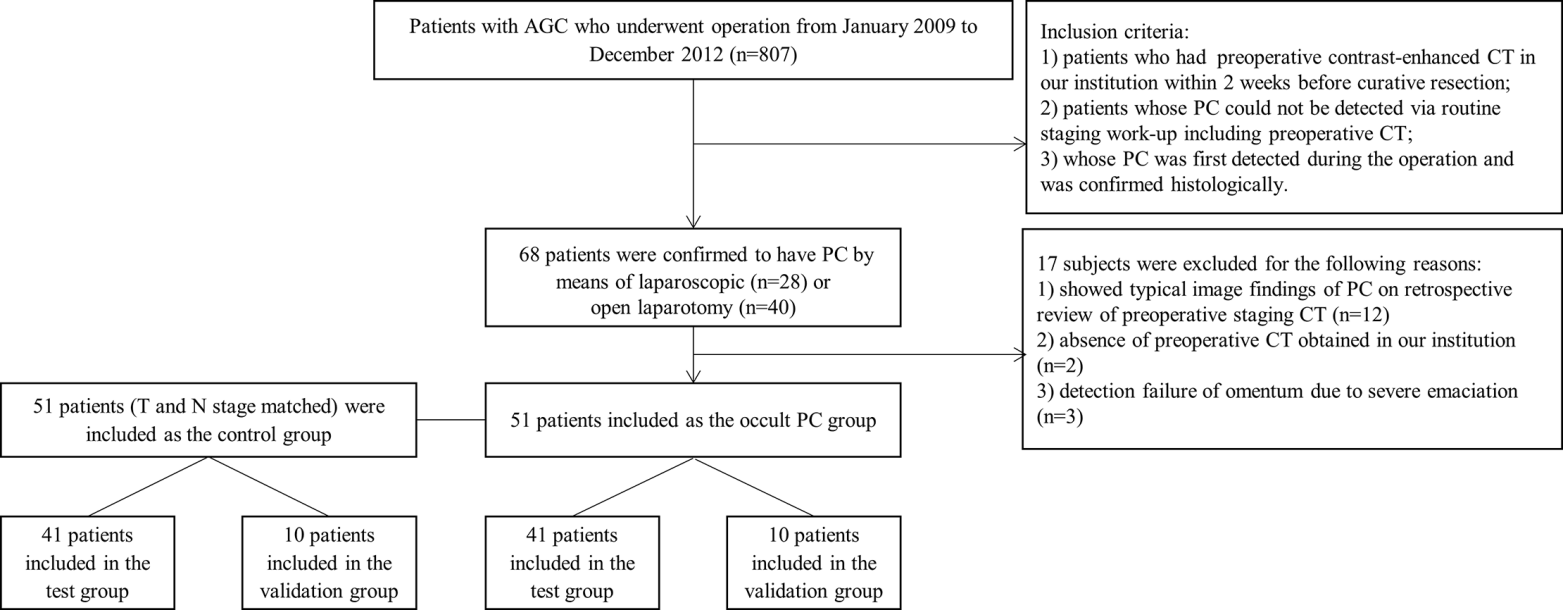
Flow chart showing the patient selection and exclusion.

807 patients with AGC who underwent operation from January 2009 to December 2012 in our hospital were identified via search of the hospital database (Fig 1). Inclusion criteria of the occult PC group were as follows: 1) patients who had preoperative contrast-enhanced CT in our institution within 2 weeks before curative resection; 2) patients whose PC could not be detected via routine staging work-up including preoperative CT; and 3) whose PC was first detected during the operation and was confirmed histologically. Exclusion criteria were as follows: 1) patients who had preoperative CT in an outside hospital, since information regarding the CT protocol was unclear; 2) patients with severe emaciation, since there was insufficient omental fat over which ROI for texture analysis could be drawn; and 3) patients who have liver cirrhosis, heart failure, or history of abdominal surgery. Among 807 patients with AGC, 68 patients were confirmed to have PC by means of laparoscopic (n = 28) or open laparotomy (n = 40) findings and histopathologic examination of either the biopsy sample from tumor implants (n = 60) or peritoneal washing cytology (n = 8). Among these 68, 12 patients showed typical imaging findings of PC on retrospective review of the preoperative staging CT [9,10]. In the remaining 56 patients, the possibility of PC was not suggested by clinical staging work up including CT; instead, PC lesions were unexpectedly detected during the operation (laparoscopic procedure in 26 patients and open laparotomy in 30 patients). After exclusion of 5 patients due to the absence of preoperative CT images obtained at our institution (n = 2) and detection failure of omentum resulting from severe emaciation (n = 3), 51 patients who had preoperative CT examination and had histologically confirmed occult PC were finally included as the occult PC group.

For the control group, 51 patients with AGC who underwent surgery from January 2011 to July 2012, and who satisfied the following criteria were included: 1) patients who had preoperative contrast-enhanced CT in our institution within 2 weeks before curative resection; 2) patients whose clinical T and N staging could be matched one-to-one to those of the patients in the occult PC group; 3) patients who showed no evidence of distant metastasis including PC at the time of CT imaging, and at least for 6 months after the surgery. Exclusion criteria of the control group were the same as those of the occult PC group.

The enrolled patients of each group were divided into the test (n = 41) and validation cohort (n = 10) according to the order of CT examination date.

### CT image acquisition

The interval between preoperative CT examination and surgery ranged from 0 to 13 days (6.8 ± 3.1 days) for the occult PC group and from 1 to 14 days (7.0 ± 3.6 days) for the control group. Contrast-enhanced CT examination was performed following injection of intravenous nonionic contrast material (2 mL/kg; iopromide, Ultravist 370: Bayer, Berlin, Germany) with power injection (Stellant D, Medrad, Indianola, PA) at a rate of 3 mL/sec. 16-, 64-, or 256-channel MDCT (Mx 8000, Brilliance 64, or iCT256; Philips Medical Systems, Cleveland, OH) were used for 61, 34, and 7 patients, respectively. The patients ingested 1.2 L of water just before the CT scan. CT scanning of portal venous phase was done after the bolus contrast media injection with a delay of 60 seconds after the aortic enhancement of 150 HU. CT scans were acquired under the following parameters: 120 kV; collimation, any of 16 x 1.5 or 64 x 0.625 or 128 x 0.625 mm; rotation time, 0.5 seconds; pitch, 1.25, 0.641, or 0.993. Tube current was automatically modulated (Dose-Right; Philips Medical Systems), and axial and coronal images were reconstructed with 4-mm thickness at 3-mm intervals by using filtered back projection.

### Clinical data

One radiologist (C.Y., with experience of 3 years in the abdominal radiology) documented the following data from the electronic medical records database: patient demographic information (age, sex), body mass index (BMI), clinical TNM stage, histological and cytological diagnoses, the date of preoperative contrast-enhanced CT imaging of the abdomen and pelvis, the date of surgery, the type of surgery received (i.e. total gastrectomy, or laparoscopic biopsy), and presence of ascites on CT.

### Texture analysis

**Fig 2:**
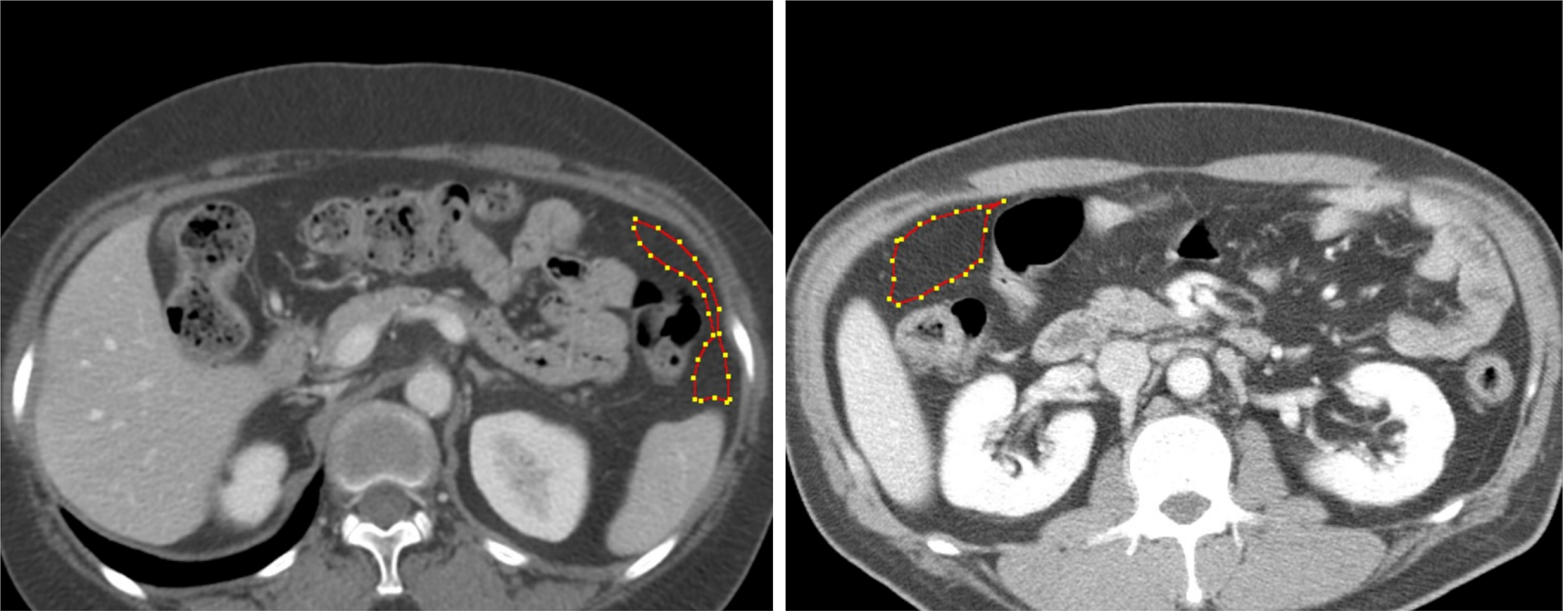
Region of interest (ROI) in texture analysis. 49-year-old male with T3N2 advanced gastric cancer (AGC) without seeding (a) showed entropy of 7.05 within the ROI. 59-year-old female with T3N2 AGC with occult seeding (b) showed entropy of 7.70, which was higher than the optimal cut-off value (> 7.141) obtained from the receiver operating characteristic (ROC) analysis.

Preoperative CT images were retrieved from the institutional image archive system, and were loaded in random order onto an independent workstation with in-house developed software for texture-analysis [13,17]. Polygonal region of interest (ROI) was manually drawn over the omental fat, on the image slice displaying the omentum in its largest cross-sectional area (Fig 2). ROI outlining was done under the consensus of two radiologists (Y.H.K., and H.Y.K., with 20 and 3 years of experience in abdominal radiology, respectively) who were blinded to the clinical and histological information. During the ROI outlining, particular attention was paid to avoid large vessels and adjacent organ. Median area and number of pixels on ROIs for the texture analysis were 265.7 cm^2^ (range, 53.0 to 1593.7), and 771.5 (range, 157 to 5128), respectively. Lesion heterogeneity within the ROI was quantified by using average (mean intensity of the gray-level distribution), standard deviation (standard deviation of the gray-level histogram distribution), kurtosis (flatness of the histogram), skewness (asymmetry of the histogram), entropy (irregularity of gray-level distribution), correlation (measurement of gray-tone linear dependencies), and contrast (local intensity variation) [11,14]. Positive skewness, lower correlation, higher entropy, standard deviation, kurtosis, and contrast, were thought to represent increased heterogeneity [11,12,14,15,18,19].

### Statistical analysis

After univariate analysis using Fisher's exact and Wilcoxon signed-rank test for the comparison of clinical and texture features between the two groups of the test cohort, conditional logistic regression analysis was performed to determine the significant independent predictor for occult PC in the test cohort. For the significant independent predictor, receiver operating characteristic (ROC) analysis for the test cohort was done, where the area under the ROC curves (AUC) were recorded and the point on the ROC curve furthest from the line of no-discrimination was considered as the optimal cut-off value. Using the optimal cut-off value, diagnostic sensitivity and specificity were determined in the test cohort. The cut-off values of the significant variables obtained from the test cohort were then applied to the validation cohort. Sensitivity and specificity for the diagnosis of occult peritoneal seeding were obtained in the validation cohort. Bonferroni correction was used to adjust *P* value for multiple comparisons. Statistical analyses were performed using MedCalc version 9.2.0.0 (MedCalc Software, Ostend, Belgium) and Stata SE version 14.0 (Stata, College Station, Tex; SAS version 9.4, SAS Institute, Cary, NC).

## Results

### Clinical features of the test cohort

41 AGC patients with occult PC (median age, 58 years; male to female ratio, 25:16) and 41 AGC patients without distant metastasis (median age, 64 years; male to female ratio, 29:12) were included as the test cohort (Table 1). Based on the clinical staging according to the 7th edition of the American Joint Committee on Cancer (AJCC) staging system, there were 2 patients with cT2N0 stage, 6 with cT3 or T4aN0, 10 with cT3 or T4aN1, 17 with cT3or T4aN2, 4 with cT4bN1, and 2 with cT4bN2, in each of the occult PC and control group for the test cohort.

**Table 1:** Univariate analysis of clinical and texture characteristics in patients with and without occult PC.

			Occult PC group (n = 41)	Control group (n = 41)	*P* value
Clinical					
	Age				0.120
		Median	58 years	64 years	
		IQR	48.0–68.0 years	54.3–72.0 years	
	Male:Female		25:16	29:12	0.49
	BMI				0.22
		Median	21.7	22.1	
		IQR	19.5–23.5	20.3–23.8	
	Ascites				
		Present	10	10	1.00
		Absent	31	31	
	Tumor grade				
		M/D	13	20	0.180
		P/D or SRC	28	21	
Texture analysis					
	Average				< 0.001
		Median	949.1	920.0	
		IQR	921.4–982.0	911.2–935.5	
	Contrast				0.038
		Median	171.1	133.9	
		IQR	119.9–221.2	111.2–158.7	
	Correlation				< 0.001
		Median	0.002	0.003	
		IQR	0.001–0.003	0.003–0.004	
	Entropy				< 0.001
		Median	7.3	7.0	
		IQR	7.1–7.5	6.8–7.1	
	Kurtosis				0.99
		Median	0.424	0.359	
		IQR	-0.291–2.064	-0.023–1.224	
	Skewness				0.110
		Median	0.522	0.322	
		IQR	0.213–1.146	0.123–0.738	
	Standard deviation				< 0.001
		Median	23.8	14.6	
		IQR	18.0–30.0	12.5–17.1	

PC indicates peritoneal carcinomatosis; IQR, interquartile range; BMI, body mass index; M/D, moderately differentiated; P/D, poorly differentiated; SRC, signet ring cell carcinoma.

In the occult PC group, 17 patients received subtotal gastrectomy; 19, total gastrectomy; 3, gastrojejunostomy; 1, open biopsy; and 1, laparoscopic biopsy. In the control group, 21 patients received subtotal gastrectomy; the remaining 20 patients received total gastrectomy.

Between the two groups of the test cohort, distribution of age, gender, BMI, presence of ascites, and tumor grade were not significantly different (Table 1).

### Texture features of the test cohort

**Fig 3:**
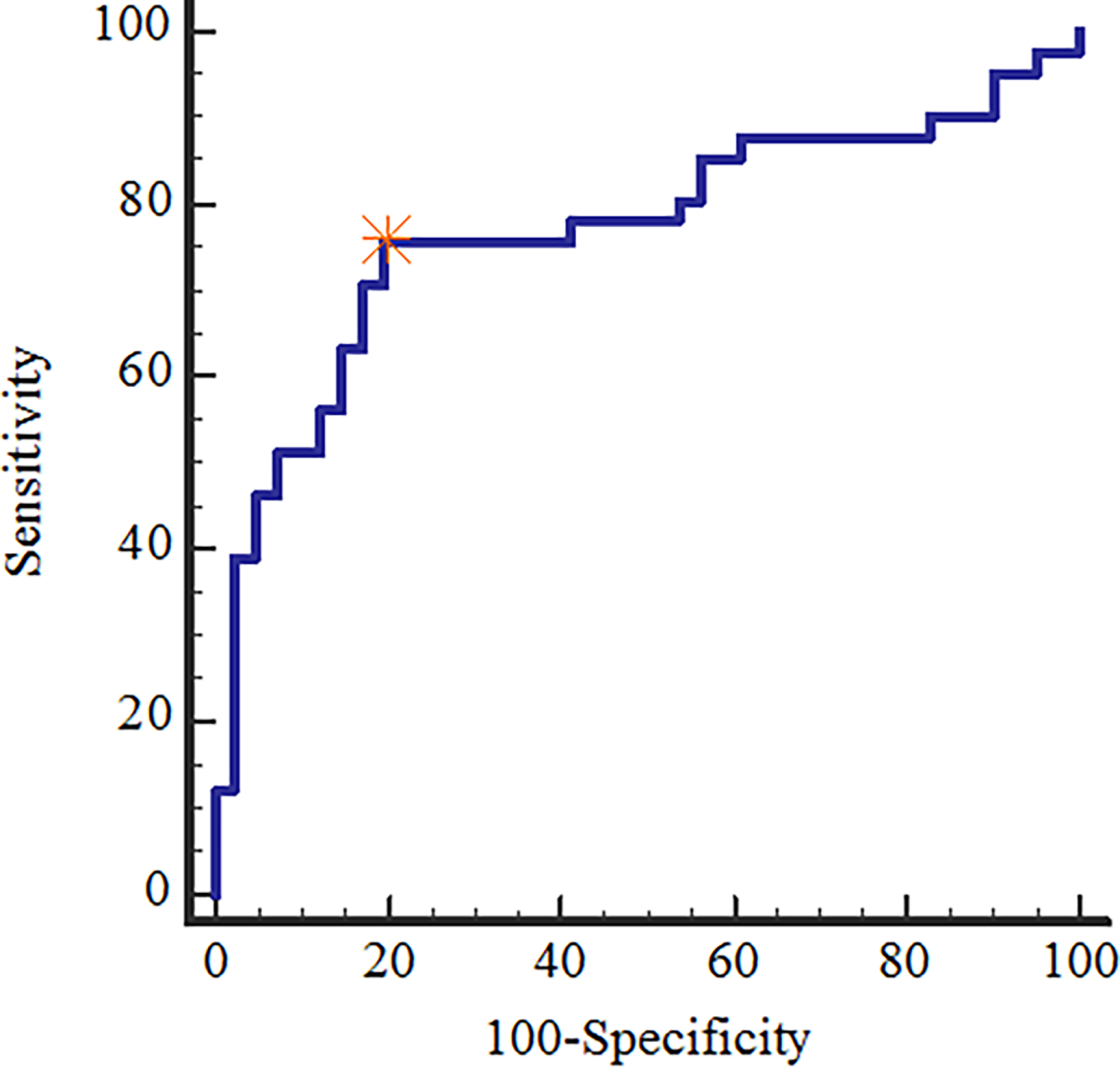
Receiver operating characteristic (ROC) curve for the entropy. Asterisk indicates the optimal cut off point (> 7.141).

The texture analysis results of the occult PC and control groups are summarized in the Table 1. The occult PC group (n = 41) showed significantly higher average, entropy, standard deviation, and significantly lower correlation (*P* value < 0.004 for all). There was no significant difference between the two groups regarding kurtosis, contrast, and skewness (Table 1). Conditional logistic regression analysis demonstrated that entropy was significant independent predictor for occult PC in the test cohort, as shown in the Table 2. ROC analysis showed that for entropy, AUC was 0.770 (0.664–0.856), with sensitivity and specificity of 75.6% and 80.5% at the optimal cut-off value (> 7.141) (Table 3 and Fig 3).

**Table 2:** Conditional logistic regression model for occult PC in the test cohort.

	Coefficient	*P* value	z	95% confidence interval of OR
Average	0.016	0.365	0.365	-0.018–0.059
Correlation	-554.666	0.550	0.550	-2373.885–1264.554
Entropy	3.824	0.026	2.23	0.458–7.190
Standard deviation	0.270	0.073	0.073	-0.025–0.566

PC indicates peritoneal carcinomatosis; OR, odds ratio

**Table 3:** Receiver operating characteristics analysis for entropy.

	AUC	Optimal cut-off	Sensitivity*	Specificity*	NPV*	PPV*
Entropy	0.768 (0.676–0.861)	> 7.14	77.5 (68.5–86.5)	76.2 (67.0–85.4)	78.1 (69.1–84.9)	75.6 (66.3–85.4)

Data in parentheses are 95% confidence interval.

*Sensitivity and specificity at the optimal cut-off value

AUC indicates are under the curve; NPV, negative predictive value; PPV, positive predictive value

### Clinical features and diagnostic performance in the validation cohort

Clinical features and diagnostic sensitivity and specificity using the cut-off value of entropy in the validation cohort are summarized in the Table 4. 10 AGC patients with occult PC (median age, 53.5 years; male to female ratio, 7:3) and 10 AGC patients without distant metastasis (median age, 54.0 years; male to female ratio, 6:4) were included as the validation cohort (Table 4). Based on the clinical staging, there were 5 patients with cT3 or T4aN0, 2 with cT3 or T4aN1, and 3 with cT3or T4aN2 in each of the occult PC and control group. In the occult PC group, 4 patients received subtotal gastrectomy; 3, total gastrectomy; 1, gastrojejunostomy; 1, open biopsy; and 1, laparoscopic biopsy. In the control group, 6 patients received subtotal gastrectomy, and the other 4 patients received total gastrectomy. There was no significant difference in the clinical features between the two groups in the validation cohort (Table 4). For the cut-off value of entropy (> 7.141), sensitivity and specificity were 80% (8/10) and 90% (9/10) respectively.

**Table 4:** Clinical features and diagnostic performance for validation cohort.

			Occult PC group (n = 10)	Control group (n = 10)	*P* value
Demographic					
	Age				0.77
		Median	53.5 years	54.0 years	
		IQR	42.0–66.0	49.0–71.0	
	Male:Female		7:3	6:4	1.00
	BMI				0.92
		Median	21.5	22.4	
		IQR	17.8–24.4	19.8–23.6	
	Ascites				
		Present	3	2	1.00
		Absent	7	8	
	Tumor grade				
		M/D	5	6	1.00
		P/D or SRC	5	4	
Diagnostic performance					
	Entropy > 7.141		8	1	
	Entropy < 7.141		2	9	
		Sensitivity	80%		
		Specificity	90%		

PC indicates peritoneal carcinomatosis; IQR, interquartile range; BMI, body mass index

M/D, moderately differentiated; P/D, poorly differentiated; SRC, signet ring cell carcinoma

## Discussion

This study used texture analysis to assess heterogeneity of the omentum of AGC patients with and without occult PC, and demonstrated that entropy was a significant independent predictor for occult PC. Using the cut-off value of entropy for the validation cohort, sensitivity and specificity for the occult PC were 80% and 90%, respectively. This result suggests that texture features obtained from routine staging CT images may be used to differentiate patients with and without occult PC, thereby preventing unnecessary surgical approach.

Multiple studies on texture-analysis for gastric cancer reported that texture analysis may effectively aid in predicting histopathological grade, overall survival, and response to neo-adjuvant therapy of gastric cancer [20–22]. Heterogeneity of texture features is known to be correlated with increased tumor heterogeneity, adverse tumor biology and decreased survival [12–15]. Tumor heterogeneity may reflect complex milieu of haphazard distribution of tumor vessels, cellularity, hemorrhage and necrosis [14,23]. There has been a study that attempted to show direct biological correlates for the texture heterogeneity; it demonstrated correlation between the tumor heterogeneity as measured via texture analysis on CT images, with markers of hypoxia and angiogenesis, such as GLUT-1 and CD34 [24]. In our study, CT texture features indicative of increased intratumoral heterogeneity were significantly associated with the presence of occult PC.

Intraperitoneal dissemination of gastric cancer is believed to occur by sloughing of cells from tumors that have invaded the serosa, which then disseminate by transmesothelial or translymphatic spread, rendering locoregional control of the disease difficult [25]. Complex lymphatic distribution, with greater number of lymphatics noted at the omentum, mesentery, diaphragm’s inferior surface, falciform ligament, and Douglas’ pouch leads to earlier PC detection in these areas [25]. This peritoneal spread eventually causes ascites, omental, mesenteric, and peritoneal seeding nodules and masses that can be observed in CT (9, 16). However, during early stage of PC, intraperitoneal micrometastases or fine nodules of subcentimeter size may not be discerned on CT and instead be detected unexpectedly during surgery; previous studies have reported limited sensitivity of CT in the detection of PC [9,10,26]. In addition to these fine nodules on fat tissue of omentum, microscopic level processes such as release of cytokines, motility factors, or matrix proteinases required for angiogenesis, may be attributable for the increased heterogeneity of the omentum of the patients with occult PC [25], though underlying mechanism remains obscure. Even though this lack of exact biological correlates for the heterogeneity is one of inherent limitations for the texture analysis, the ease of obtaining the texture information from the routinely acquired CT images without additional imaging or procedure, and accumulating data showing correlation between heterogeneity and adverse tumor biology, are major advantages of the technique. Management of PC in AGC patients is getting more sophisticated and showing promise in prolonging the life expectancy, making timely diagnosis of PC crucial. Texture analysis on CT image holds clinical significance, as it may contribute to the diagnosis of PC in AGC.

Majority of previous studies used texture analysis to measure heterogeneity within the ROI drawn over a focal lesion such as tumor or metastatic lymph node, whereas the current study tried to quantify texture of omentum without any overt mass formation on CT [12–16,19]. Similar approach was attempted for the seemingly normal area of the liver parenchyma in patients with extrahepatic metastases, or known hepatic metastasis elsewhere [27–29]. In a previous study by Ganeshan *et al*., hepatic entropy measured at an apparently disease-free area of the liver was significantly lower for the patients with hepatic metastases compared to those without hepatic metastases [27]. The authors tried to explain this finding by suggesting that angiogenic changes caused by hepatic metastases may lead to increased arterial perfusion, resulting in vascular dilatation that may be seen as increased uniformity of the remaining normal liver of the patients with hepatic metastases. The study concluded that texture features, compared to simple attenuation measurements, could reflect tumor-related changes of hepatic parenchyma more sensitively [27]. These previous results, together with the findings of our study, suggest that texture analysis could be successfully applied to, and produce meaningful result for non mass-forming region such as omentum or an organ’s parenchyma.

There are several limitations of this study. First, there was bias inherent to the retrospective study design, such as selection bias. Second, ROI for the texture analysis was drawn over the omentum, whereas peritoneal carcinomatosis detected by surgeons was not necessarily limited to a nodule discovered in the omentum, but included anywhere including the cul-de-sac, mesentery or peritoneum. However, even in patients whose PC nodule was detected elsewhere, there is high probability for these patients to have harbored microscopic metastatic foci at the omentum as well. Third, patients of this study were recruited over a few years, during which there have been changes of practice caused by advances of CT scanner and accumulation of new knowledge. The 7th edition of the American Joint Committee on Cancer (AJCC) staging system regards intraperitoneal cancer cell detection from peritoneal washing cytology as equivalent to peritoneal carcinomatosis [30]. Some of the recruited patients of this study had received surgery before peritoneal washing cytology was adopted in our hospital; there is possibility that some patients with occult PC lesions that could not be detected by surgeons were erroneously categorized to the control group, especially during the period when cytology was not used. To minimize this potential error, we included patients in the control group only if there was no evidence of distant metastasis during follow-up at least for 6 months after the surgery. Considering the aggressive and rapid progression of patients with peritoneal carcinomatosis, possibility of these patients to have harbored micro-PC during the time of surgery would be very low [31]. Fourth, emaciated patients were excluded from the study population due to lack of omentum over which ROI could be drawn. For the current study, the number of exclusion was relatively small (3 out of 58), but this problem of ROI outlining may act as a significant drawback in analyzing the omentum of gastric cancer patients who are not infrequently cachectic. Fifth, since 2 radiologists drew ROI for all cases based on consensus, inter-observer agreement was not investigated in this study. Although there was a previous study that reported that inter-observer agreement in texture analysis for gastric cancer was generally good [22], this study involved drawing ROI over gastric cancer; it is unknown whether inter-observer agreement for drawing ROI over omentum, which is an uncircumscribed area without boundary, will be comparable to that for drawing ROI over any mass or wall thickening. Lastly, the cut-off values for texture features reported in our study may not be directly applicable to images from the other CTs, since varying CT acquisition parameters may influence the texture features. Nonetheless, the results of this study will be of great asset to the ongoing studies on texture analysis and attempts towards its clinical application.

In conclusion, management of PC in AGC patients is getting more sophisticated and showing promise in prolonging the life expectancy, making timely diagnosis of PC crucial. For AGC patients whose PC cannot be detected with routine imaging such as CT, texture analysis may be a useful adjunct for the prediction of occult PC.

## Supporting information

S1 File Data of the test cohort. (XLSX)
